# Reactions and perspectives of medical students on workplace violence during clinical training in Ardabil, Iran, 2020

**DOI:** 10.1186/s12909-023-04426-7

**Published:** 2023-06-13

**Authors:** Saber Sadeghi, Atefeh Shadman, Afrouz Mardi, Daniel Hackett

**Affiliations:** 1 Medical faculty, Ardabil University of Medical Science, Ardabil, Iran; 2 Specialist of community and preventive medicine, Ardabil University of Medical Science, Ardabil, Iran; 3 Department of Public Health, School of Health, Ardabil University of Medical Science, Ardabil, Iran; 4 Physical Activity, Lifestyle, Ageing and Wellbeing Faculty Research Group, School of Health Sciences, Faculty of Medicine and Health, The University of Sydney, Lidcombe, NSW Australia

**Keywords:** Workplace violence, Reaction, Perspective, Medical students

## Abstract

**Background:**

Workplace violence continues among medical students in training. This study aimed to determine the reactions and perspectives of medical students against workplace violence during clinical training in Ardabil University of Medical Sciences in Iran, 2020.

**Materials:**

This descriptive cross-sectional study was conducted on 300 medical students from April to March 2020, in the Ardabil university hospitals. Students with at least one year training in the university hospitals were eligible to participate. Data was collected via questionnaires administered in the health ward. Data was analyzed through SPSS 23 software.

**Results:**

Most respondents had experienced workplace violence in the form of verbal (63%), physical (25.7%), racial (23%) and sexual (3%) violence during clinical training. Men were the aggressors during violence of a physical (80.5%), verbal (69.8%), racial (76.8%) and sexual (100%) nature (p < 0.001). When encountered with violence, 36% of the respondents did not take any action and 82.7% of respondents failed to report the incident. For 67.8% of respondents that did not report of violence incident, this procedure was deemed pointless, while 27% of respondents considered the violent incident insignificant. The main reason for workplace violence was perceived to be a lack of awareness of people about staff duties (67.3% of respondents). According to 92.7% of respondents personnel training was the most important factor in preventing workplace violence.

**Conclusions:**

The findings suggest that the majority of medical students during clinical training in Ardabil Iran (2020) have been exposed to workplace violence. However, most students did not take any action or report the incident. Targeted personnel training, increase awareness of workplace violence, and encouragement of reporting these incidents should be promoted to reduce violence to medical students.

## Background

The phenomenon of violence has been one of the most perplexing issues in the social life that continues to this day [1]. Workplace violence (WPV) is defined as instances of staff being harassed, threatened, or attacked at their workplace (WHO, 2002) [2]. WPV is the third leading cause of occupational injury-related death in the United States and the second major cause of female workplace mortality [3]. Numerous studies indicate that staff members in health fields and hospitals face more WPV compared to staff in other fields [4, 5]. According to the International Council of Nurses, health care workers may face more violence than prison guards or police officers [6]. WPV prevalence varies significantly across countries and environments [7]. For example, according to the U.S. Bureau of Labor Statistics, health care workers sustained 73% of violence-related injuries and morbidities in 2018 [8]. This figure is reportedly 56.4% in China [9] and > 85% in Iraq [10]. In Iran, over half of the medical staff are thought to be subjected to WPV [11]. WPV may manifest itself in various ways, including physical, emotional, sexual or verbal [12], and may involve every occupational group [13].

International studies estimate that WPV occurs at a rate of 60 to 70% among medical staff [14–16].

Physicians were more likely to be exposed to serious WPV than nurses [9]. As for medical students, clinical workplace-based teaching may predispose them to violence due to direct contact with patients and their relatives [17, 18]. In Iran, the prevalence of physical and verbal violence among medical students has been reported to be 4.5% and 59%, respectively [19]. A study in China reported that WPV may drive medical students to leave the health labor market, or seek jobs with lower risk, and not pursue practicing medicine [20]. In other study on medical and nursing students in Israel, most students reported experiencing verbal abuse during their clinical training. This previous study reported that WPV caused some students (14.6%) to consider leaving the profession, and alarmingly, most students did not report experiencing physical violence [21].

Various studies show that violence in the hospital system is highly prevalent and most widespread in psychiatric wards, emergency wards, waiting rooms, and the elderly unit [22]. WPV can have a devastating impact on medical students and staff, including decreased self-esteem, job satisfaction and quality of life, quitting a job, and increased anxiety and stress in health caregivers [15, 23, 24]. Additionally, violence has a detrimental effect on the commitment of health caregivers, resulting in a decreased quality of patient care [25]. On the other hand, workplace violence imposes costs on staff in the form of medical and psychiatric care, missed workdays, decreased productivity, compensation, and litigation [22, 26]. Furthermore, violence could have a negative effect on students’ academic performance and achievement due to disrupting cognitive development, and impairing their ability to concentrate [27].

The negative effects of violence on well-being and productivity of medical students in training is well documented. Despite this, it appears that these behaviors continue, which may negatively impact the health and well-being of medical students [28]. This study aimed to determine the reactions and perspectives of medical students against workplace violence during clinical training in Ardabil University of Medical Sciences in Iran, 2020.

## Methods

A cross-sectional study designed used to investigate reactions and perspectives of medical students against workplace violence. Trainee students and interns (6th and 7th year medical students) attending teaching hospitals of Ardabil university of medical sciences participated in this study from April to March 2020. Ardabil is in northwest Iran and has an estimated population of 1,300,000 people (2016 census) who speak Azeri. It covers an area of approximately 18,000 km^2^ or about 1% of Iran’s total area [29]. Ardabil has 5 teaching hospitals with relevant specialized services, including Imam Reza Hospital (urology, ophthalmology, dermatology and pediatric surgery), Alavi Hospital (gynecology and obstetrics, neonates and neurology), Fatemi Hospital (psychiatry, trauma and burns), Imam Khomeini Hospital ( general diseases of adults) and Bo Ali Hospital (children’s diseases).The total number of medical students from the all teaching hospitals was approximately 600; thus, the sample size was determined to be 234, by using the Krejcie Morgan table. Finally, 300 students were included in the study using stratified random sampling to avoid sample dropout. In this way, the required number of samples was randomly selected from each hospital in proportion to the number of students in that hospital. All medical students who had trained at least one year in the university hospitals, met the inclusion criteria. Exclusion criteria were dissatisfaction with participation in the study. All participants provided written consent and were informed of the study’s topic and data collection method. Participant information was guaranteed to be kept confidential and anonymous. This study was approved by the ethics committee of Ardabil University of Medical Sciences under code IR.ARUMS.REC.1398.312.

Data was collected using a demographic questionnaire and the standard WPV questionnaire in the health ward, developed by the World Health Organization, the International Labor Organization, and the International Council of Nurses (ICN). Briefly, this questionnaire contains 53 questions divided into four sections, 15 questions addressing physical violence, 13 questions on verbal abuse, 12 questions on sexual harassments, and 12 questions on racial harassments. At the end of the questionnaire, three open-ended questions were included: (1) “What did you do when you were abused?” (2) “What are your thoughts on the effective factors in violence?” and (3) “How can violence in the ward be prevented?” [30]. Fallahi et al. established the reliability of this questionnaire’s Persian version by using a correlation coefficient of 0.73 [31].

### Statistical analysis

Data analysis was performed using SPSS 23 software. Descriptive statistics (frequency and percentage) and inferential statistics (chi-square and independent t-test) were used to determine the relationship between variables.

## Results

The mean age of respondents was 24.9 ± 2.1 years, with females accounting for a greater proportion of participants (59.7%). Most respondents were sixth year medical students (51%), single (84.3%) and of Muslim (Shiite) faith (96.3%). Turks (Azari) represented 87.7% of participants, followed by7.7% being Persian, 2.3% being Kurds, and the remainder from other ethnicities.

The type of violence encountered by medical students included verbal (63%), physical (25.7%), racial (23%) and sexual (3%) (Table 1). Men were the aggressors in 100% of sexual harassment, 80.5% of physical violence, 76.8% of racial harassment and 69.8% of verbal abuse (p < 0.001). In all forms of violence, the aggressors involved the patient’s relatives, the patient, colleagues, non-colleague staff, and others, respectively, while relatives committed 100% of sexual harassment. (p < 0.005).

**Table 1:** Characteristics of workplace violence in medical students

Variables	Verbal Abuse n (%)	Sexual Harassment n (%)	Physical Physical n(%)	Racial Harassment n(%)	p value
Facing violence Yes	189(63)	9(3)	77(25.7)	69(23)	
**The gender of the aggressor** Male Female	132(69.8) 57(30.2)	9(100) 0(0)	62(80.5) 15(19.5)	53(76.8) 16(23.2)	P < 0.001
**Aggressor** Patient Relatives of the patient Colleagues Employees Others	51(27) 75(39.7) 32(16.9) 16(8.5) 15(7.7)	0(0) 9(100) 0(0) 0(0) 0(0)	29(37.6) 41(53.2) 3(3.9) 1(1.3) 3(3.9)	19(27.5) 23(33.3) 11(15.9) 13(18.8) 3(4.3)	P < 0.005
**Time of violence** Morning (8:00–14:00) Evening (14:00–20:00) Night (20:00–8:00)	93(49.2) 32(16.9) 64(33.9)	3(33.3) 3(33.3) 3(33.3)	26(33.8) 12(15.6) 39(50.6)	56(81.1) 10(14.5) 3(4.3)	X^2^ = 61.3 P < 0.003
**Aggressor’s mean age**	38.44 ± 4.7	38.25 ± 3.9	35.47	37.52	
**Violence ward** Surgery Internal Psychiatry Trauma Pediatrics Gynecology Others	16(8.5) 77(40.7) 35(18.5) 27(14.3) 2(1) 8(4.2) 24(12.7)	3(33.3) 3(33.3) 3(33.3) 0(0) 0(0) 0(0) 0(0)	5(6.5) 17(22.1) 30(38.9) 8(10.4) 0(0) 2(2.6) 15(19.5)	0(0) 46(66.7) 20(29) 3(4.3) 0(0) 0(0) 0(0)	X^2^ = 75.2 P < 0.001
**Days of violence** Saturday Sunday Monday Tuesday Wednesday Thursday Friday Most days	24(12.7) 11(5.8) 15(7.9) 10(5.3) 16(8.5) 15(7.9) 8(4.2) 89(47.1)	0(0) 3(33.3) 0(0) 3(33.3) 0(0) 3(33.3) 0(0) 0(0)	20(26) 10(13) 3(3.9) 4(5.2) 4(5.2) 12(15.6) 16(20.8) 8(10.4)	4(5.8) 4(5.8) 2(2.9) 4(5.8) 9(13) 6(8.7) 5(7.2) 35(50.7)	X^2^ = 6.34 P = 0.175

Physical violence was most prevalent (50.6%) during the night shift. Nevertheless, the morning shift had the highest rate of verbal abuse (49.2%) and racial harassment (81.1%), while sexual harassment was consistent across all three shifts (33.3%). This difference was statistically significant (p < 0.003).

The mean age of aggressors ranged between 35.5 and 38.3 years for physical violence and sexual harassment cases, respectively. The psychiatry ward reported the highest rate of physical violence (38.9%), while the internal ward reported the highest rates of verbal abuse (40.6%) and racial harassment (66.7%). This difference was statistically significant (p = 0.001).

Study of occurrence of violence in various days of week revealed.

that the highest rate of physical violence (26%) occurred on Saturdays; however, the rate of verbal abuse (47.1%) and racial harassment (50.7%) were high on most days. There was no statistically significant difference between types of violence and days of the week (χ2 = 6.34, p = 0.175 (Table 1).

Study of the student’s reactions to the violence showed that the most respondents (36%) took no action, 26% defended themselves, 25.3% invited the aggressor to calm down, and only 3.0% asked for compensation (Table 2). Additionally, the study’s findings indicated that 17.3% of respondents reported the violence, while 82.7% of respondents failed to report the violence. The report was deemed pointless by 67.8% of respondents who did not report the incident, and the issue was insignificant according to 27% of respondents. There is no system or procedure in place for reporting WPV, according to 80.6% of respondents. Furthermore, 83.8% of respondents reported no one had taken action to address the violence thus far, and 69.7% of respondents were completely dissatisfied with the follow-up of the violence cases.

**Table 2:** Reactions of medical students to workplace violence

Reaction	N	%
Failed to act	108	36
Invited the harasser to calm down	76	25.3
Informed friends and family	3	1
Informed colleagues	10	3.3
Defended myself	78	26
Pretended the incident did not take place	9	3
Sough a counselor	3	1
Asked for help	8	2.7
Complained	4	1.33
Asked for compensation	1	0.3

Regarding the causes of violence, 67.3% of respondents stated that the “lack of awareness of the people about our duties” was the cause of the violence. In addition, 92% of respondents stated that they had never been trained in violence prevention (Table 3). In terms of effective factors for preventing of WPV, 92.7% of respondents cited personnel training as the primary factor, while 65.3% of respondent believed that the police force is be an effective preventive action (Table 4).

**Table 3:** Perspectives of medical students about factors contributing to violence

Factors	n	%
People’s lack of awareness of our responsibilities	202	67.3
Patients’ use of psychotropic medications or alcohol	92	30.7
Delay in receiving help	83	27.7
Low number of people in the ward	48	16
Grouping of high-risk patients with each other	35	11.7
Patient death	47	15.7
Patient legal issues	34	11.3
Lack of patient visits	11	3.7
The interval between admission time and the patient’s definitive diagnosis	38	12.7
Extended stay of the patient in the ward after discharge	12	4
Lack of training programs to prevent violence	276	92
Inadequate security measures	30	10

**Table 4:** Perspectives of medical students about factors preventing violence

Factors	N	%
Inhibiting rules and regulations	120	40
Law enforcement action	196	65.3
Motivational actions (encouragement and punishment) of aggressors of violence	45	15
Staff training	278	92.7
Compensation of damages by management	9	3
Other cases	15	5

## Discussion

Findings from this study indicated that most medical students experienced at least one type of workplace violence during clinical training in Ardabil, Iran (2020). These results corroborate with previous studies, in Germany [32], Syria [33] and Turkey [18]. Moreover, the most common type of violence was verbal abuse, while the least common type was sexual harassment. Several studies agree with these findings [18, 31, 34]. It is highly plausible that reporting of real cases of violence is usually low due to a cultural norm or fear of possible consequences. Especially in sexual harassment cases, therefore the occurrence of sexual harassment in present study is likely to be underreported. Greater exploration and discussion is required in medical placement programs to determine appropriate measures to break this vicious cycle of silence.

Consistent with the findings from Yousefi et al., [35] and Karakas et al. [36], the majority of violence reported in the present study were committed by men and patient’s relatives. Kuhestani et al. observed that a significant proportion of physical violence was committed by the patients themselves, while most verbal abuses were committed by the patient’s relatives [37]. Patients may be less likely to commit violence against medical staff and health care workers because of their illness, age, and physical condition. Consequently, relatives of patients may resort to violence because of anger, worry, dissatisfaction, or financial intentions (demand for compensation).

The present study’s findings indicated that the night shift had the highest rate of physical violence, while the morning shift had the highest rate of verbal abuse and racial harassment. Sexual harassment, on the other hand, was consistent across all three shifts. According to a study conducted by Nourizadeh et al. (2014), the night shift was the epicenter of violence in all its representations [38]. The results showed that students working night shifts with less support and care were more aggressed and those students working morning shifts were more likely to face verbal abuse, most likely due to the high number of patients and crowded hospital.

According to this study, the highest rate of violence was observed in the hospital’s psychiatry and internal wards. In contrast to our study findings, Kumar et al. showed that the highest level of violence occurred in the obstetrics and gynecology wards in India [39]. Furthermore, a systematic review of eight studies, found that the highest rates of violence occurred in emergency, internal, and surgery wards [40]. Another significant finding of our study was that most of the students had no reaction against violence. This finding also corroborates with previous research findings [18, 31]. In the study conducted by Fallahi et al. (2013), the most frequent response against violence was to invite the aggressor to calm down, defend themselves, and seek assistance [31]. Teymourzadeh et al. (2009) found that most subjects responded to violence by inviting the aggressor to calm down [41]. As a result, it can be hypothesized that many medical students have accepted workplace violence as a part of their profession.

Another significant finding of the present study was that most students who were abused (82.7%) failed to report the incident. They believed that reporting the incident was “uselessness,” would be considered an “insignificant incidence,” and there was an “absence of a system or instruction for reporting violence”. This finding was also consistent with the results of other studies [18, 31, 41]. The factors preventing medical students from reporting an incident of violence appears to be deeply embedded in the culture of medicine, and lead to negative consequences.

A further finding of current study was students’ perspectives on the factors that contribute to WPV. The majority of students stated that the cause of violence against them was a “lack of people’s awareness of our responsibilities” and a “lack of education about violence control”, which was consistent with findings from other studies [37, 42]. In the other study, most of the respondents stated that the social image of health care providers is one of the main causes of violence against them [43]. Many people mistakenly believe that workplace violence is part of health workers job [44]. However, medical students in Turkey were dissatisfied with the role of media in this issue and believed that the frequent presentation of violent incidents does not lead to increased public awareness or rejection of violence completely, but rather increases the possibility of acceptance of violence by medical students [18]. While the other causes of violence were pointed out to be lack of treatment facilities, low quality of care, patients’ resistance to some treatment measures, pain and suffering imposed on patients due to the lack of expertise of students, high health care costs, lack of faith in the judicial system, poverty and illiteracy [43, 45].

Finally, when medical students were asked about the most effective factors in preventing WPV, staff training and the presence of law enforcement were cited as the primary factors. These responses are similar to other studies, in addition to other factors linked to violence such as insufficient staffing, drug and alcohol consumption by patients or companions, a lack of security facilities and patient death. The law and trust in the judicial system have also been mentioned in other studies as reasons for preventing violence, which confirms the results of the present study [43]. Increased guarding staff, psychiatric counselors, and psychologists onwards, increased rest hours, reduced physician workload, Commitment of students to update their awareness and knowledge about workplace violence and a well-organized training program are also considered anti-violence measures [31, 35, 46, 47].

### Research limitations

This study was limited because the findings relied on self-reporting, which may not entirely correspond to reality.

## Conclusion

In conclusion, this study showed that the prevalence of workplace violence especially verbal abuse is high among medical students. While, most of them had no action in facing with violence and failed to report it. The majority of students consider the lack of public awareness about the students’ duties and staff training as the main factors in creating and preventing violence, respectively. The need for awareness and training programs to counteract workplace violence for medical students has become more necessary than ever and may be more useful if it was in the curriculum. In addition, it is recommended to pay more attention to the factors affecting the creation and prevention of violence from the perspective of students and follow-up of reported cases.

## Data Availability

The datasets used and/or analyzed during the current study are available from the corresponding author on reasonable request.

## Abbreviations

WPV: Workplace violence
